# The effects of spinach-derived thylakoid supplementation in combination with calorie restriction on anthropometric parameters and metabolic profiles in obese women with polycystic ovary syndrome: a randomized, double-blind, placebo-controlled clinical trial

**DOI:** 10.1186/s12937-020-00601-4

**Published:** 2020-08-11

**Authors:** Fatemeh Pourteymour Fard Tabrizi, Mahdieh Abbasalizad Farhangi, Maryam Vaezi, Salar Hemmati

**Affiliations:** 1 Nutrition Research Center, Tabriz University of Medical Sciences, Tabriz, Iran; 2 Drug Applied Research Center, Tabriz University of Medical Sciences, Attar-neishabouri Ave, Golgasht St, Tabriz, 5165665931 Iran; 3 Women’s Reproductive Health Research Center, Tabriz University of Medical Sciences, Tabriz, Iran; 4 Department. of Obstetrics and Gynecology, Alzahra Teaching Hospital, Tabriz University of Medical Sciences, Tabriz, Iran; 5 Drug Applied Research Center, Tabriz University of Medical Sciences, Tabriz, Iran

**Keywords:** Polycystic ovary syndrome, Obesity, Thylakoid, Spinach-derived thylakoid

## Abstract

**Background:**

There is a promising outlook regarding the potential effect of spinach-derived thylakoids in the management of obesity and its associated metabolic disturbances. This research aimed to evaluate the effects of spinach-derived thylakoids supplementation combined with a calorie-restricted diet on anthropometric and metabolic profiles in obese women with the polycystic ovary syndrome (PCOS).

**Methods:**

In a 12-week double-blind placebo-controlled randomized clinical trial, 48 females with obesity and PCOS were randomly allocated into either intervention (5 g/day thylakoid) or placebo (5 g/day cornstarch) groups along with calorie-restricted diets. Anthropometric measures, physical activity levels, dietary intakes, insulin resistance markers, as well as serum levels of insulin, fasting blood glucose (FBG), non-esterified fatty acids (NEFA), and sex hormones including dehydroepiandrosterone sulfate (DHEAS), follicle-stimulating hormone (FSH), luteinizing hormone (LH), sex hormone-binding globulin (SHBG), and free androgen index (FAI) were evaluated pre-and post-intervention.

**Results:**

After the 12-week intervention, there were significant decreases in weight (− 6.97 ± 0.52 vs. -3.19 ± 0.72 kg; *P* < 0.001), waist circumference (− 7.78 ± 2.50 vs. -3.73 ± 1.40 cm; *P* < 0.001), fat mass (− 5.19 ± 0.53 vs. -1.36 ± 0.39 kg; *P* < 0.001), and insulin levels (− 5.40 ± 1.86 vs. -1.19 ± 0.85 μU/mL; *P* < 0.001) in the spinach-derived thylakoid group compared to the placebo group. Furthermore, insulin resistance markers and serum levels of testosterone decreased significantly in the thylakoid group compared to the placebo group (*P* < 0.05). The changes in other parameters did not show significant differences between the two groups.

**Conclusions:**

Spinach-derived thylakoid supplementation resulted in more favorable improvements in anthropometric indices and insulin sensitivity compared to the calorie restriction alone.

**Trial registration:**

The study was approved by the Ethics Committee of Research Vice-chancellor of Tabriz University of Medical Sciences, Tabriz, Iran, and was registered in the Iranian Registry of Clinical Trials (registration ID: IRCT20140907019082N9).

## Background

Polycystic ovary syndrome (PCOS), as the most common endocrine disorder in reproductive-aged women, is a multisystem disorder and a heterogeneous condition with a wide range of signs and symptoms [1]. It affects 5–20% of premenopausal women, depending on the population screened and the diagnostic criteria [2]. Based on the Rotterdam criteria, it was estimated that about 19.5% of Iranian women were affected with PCOS [3]. Mainly, the PCOS status influences reproductive, metabolic, social, and psychological facets of affected women’s lives [4]. Women with PCOS are characterized by irregular menstrual cycles, androgen excess symptoms (i.e., androgenic alopecia, hirsutism, and acne), obesity, and insulin resistance (IR) [5]. Hyperandrogenism and IR are chiefly involved in the pathogenesis of PCOS [6]. IR and concomitant compensatory hyperinsulinemia, are prevalent in PCOS; it is estimated that 50 to 75% of women with PCOS have hyperinsulinemia [7]. Obesity and overweight prevalence in PCOS was reported to be 61% in a meta-analysis conducted in 2012 [8]. There are complicated and interrelated interactions between IR, hyperandrogenism, changed ratio of the release of luteinizing hormone (LH) to follicle-stimulating hormone (FSH), and obesity; these interactions impair metabolic and hormonal profiles in obese women PCOS [5]. IR exacerbates hyperandrogenism through increased ovarian androgen production and decreased sex hormone-binding globulin (SHBG) production. On the other hand, increased production of androgens leads to IR and hyperinsulinemia [6, 9]; also, obesity can further exacerbate IR, hyperandrogenism, and menstrual irregularities [8]. There is no particular remedy or cure for PCOS, and therapeutics for PCOS management have typically focused on targeting the presentation of symptoms [10]. The first published international evidence-based guidelines for PCOS management, in 2018, emphasized the importance of interdisciplinary care for women with PCOS and highlighted a healthy lifestyle, including a focus on a diet to attain and/or maintain a healthy weight to improve hormonal health and quality of life in women with PCOS [10]. Several studies have reported that a 5 to 10% weight loss can ameliorate clinical outcomes including, risk factors for cardiovascular disease, type 2 diabetes, and endocrine and reproductive parameters in PCOS sufferers [11]. Calorie-restricted diets are the first line of obesity management [12]. It has been reported that calorie-restricted diets (350–1000 kcal/day deficit) have considerable beneficial effects on the loss of body weight and fat mass, as well as amelioration of insulin sensitivity and menstrual cycle; hence, should be recommended as the first-line therapy in PCOS women [13, 14]. Further, there has been increasing interest in the use of phyto-nutraceuticals as complementary therapies to prevent and manage PCOS related complications [15]. Recent studies have shown that thylakoids, as chloroplast membranes in green leafy vegetables, extracted from spinach have anti-obesity effects [16, 17]. The beneficial effects of thylakoid intake on obesity and its related metabolic disturbances including, reduction of blood lipids and glucose and improvement of insulin resistance, are supported by some evidence [18, 19]. Considerable reduction in ratings of hunger and desire for high-sugar foods as well as salty and high-fat foods is the most common effect that has been demonstrated by studies [16–18]. In a clinical trial, the supplementation of obese women with 5 g/day thylakoid (as a glass of blueberry juice containing 5 g thylakoid) for 3 months, resulted in significant decreases in body weight, waist circumference, low-density lipoprotein cholesterol (LDL-C), fasting blood glucose (FBG), and fasting insulin [18]. To the best of our knowledge, there is no study investigating the effects of spinach-derived thylakoid supplementation with or without a calorie-restricted diet on the hormonal parameters and metabolic status in obese women with PCOS. Therefore, the evaluation of the effects of thylakoid consumption in combination with a calorie-restricted diet on anthropometric parameters and metabolic profiles in obese women with PCOS was the purpose of the present study.

## Methods

### Study design and participants

This study was a randomized, double-blind, placebo-controlled clinical trial, conducted from September 2018 through May 2019, in Tabriz, Iran. This clinical trial was registered in the Iranian Registry of Clinical Trials (http://www.irct.ir; registration no. IRCT20140907019082N9). The target population of the present study was obese women with PCOS who were recruited from Sheykholrayis Polyclinic and the gynecology and infertility clinics of Alzahra Hospital in Tabriz, Iran. All patients were taking oral contraceptive pills (OCPs) as a routine medical therapy under the supervision of their physician. Forty-eight obese women (body mass index (BMI): 30–40 kg/m^2^) aged 20–45 years were enrolled. They were diagnosed with PCOS based on Rotterdam criteria [20]. Two of the following three features have to be present for the PCOS diagnosis: (i) oligomenorrhea with eight or fewer menstruations in the previous 12 months or amenorrhea, (ii) clinical and/or biochemical signs of hyperandrogenism, and (iii) polycystic ovaries on ultrasound examination (> 12 follicles, 2 to 9 mm in diameter and/or increased ovarian volume > 10 mL). The diagnosis of PCOS also required that there was no evidence of thyroid disease, adrenocortical dysfunction, or hyperprolactinemia (prolactin > 30 mg/mL). The main exclusion criteria for participation in this study were: menopause, or pregnancy, or lactation; smoking or being exposed to cigarette smoke (passive smoking); having co-morbidity with other gynecologic or endocrine disease, or hepatic, renal, or cardiovascular disease, diabetes and/or impaired glucose tolerance; taking any nutritional or herbal supplements during two months prior to the study, using ovulation induction agents or drugs affecting metabolic or insulin status such as statins, thiazolidinediones, corticosteroids, insulin, anti-obesity and anti-diabetic drugs (i.e., Metformin, sulfonylureas,..). If the patients had adopted a diet and/or a specific physical activity program, or any changes in medications, or experienced any detrimental events during the study, they were withdrawn from the clinical trial.

### Ethics approval and consent to participate

The study was conducted according to the Declaration of Helsinki guideline and approved by the ethics committee of research vice-chancellor of Tabriz University of Medical Sciences, Tabriz, Iran (Ethics code: IR.TBZMED.REC.1397.447); After being given a full explanation of the study procedures, written informed consent was obtained from all patients.

### Sample size

The sample size for the study was calculated based on the results mean (standard deviation; SD) for FBG as reported by Stenblom et al. [21], with a confidence interval (CI) of 95%, and power of 90% in two-sided tests using power analysis and sample size software (PASS; NCSS, LLC, US) version 15, the sample size was 21 per group, which was increased to 24, considering a probable 15% dropout rate.

### Study protocol

The study participants were randomly allocated into one of the two experimental groups (1:1), by the use of the Random allocation software (RAS) and randomized block procedure of size 2 (age (< 33 vs. ≥33) and BMI (< 35 kg/m^2^ vs. ≥35 kg/m^2^)). Randomization and allocation were concealed to the researchers and participants until the final analyses were completed. The eligible subjects were assigned to receive 5 g/day of thylakoid-rich spinach extract or matching placebo as 5 g/day of raw corn starch (one sachet, 30 min before lunch) for 12 weeks. The sachets were completely identical in all other aspects. The primary outcomes were changes in anthropometric measurements and metabolic status. At first, demographic and clinical questionnaires were completed for all participants. They were asked to complete a 3-day food record questionnaire including, two weekdays and one weekend day in the week just prior to the study and during weeks 6 and 12. The International Physical Activity Questionnaire - short form (IPAQ-SF) was also used to assess the physical activity level, at baseline, week 6, and week 12. Next, anthropometric measurements, bioelectric impedance analysis (BIA), and blood pressure measurements were performed. After 12-h overnight fasting, blood was taken from each patient for biochemical evaluations before and after the intervention. The participants were asked to keep their regular medication (i.e., OCP) and usual levels of physical activity throughout the study period. They were also advised to inform the researchers of any changes in their medical therapy program and any adverse effects of the supplements.

### Intervention protocol

Participants were randomly recruited to a 12-week intervention arm consisting of 5 g/day of thylakoid-rich spinach extract powder + low-calorie diet (*n* = 24) or to a control arm of 5 g/day powdered raw cornstarch as placebo + low-calorie diet (*n* = 24). The choice of 12-week intervention duration and a dose of 5 g/ day thylakoid was based on previously reported beneficial effects of thylakoid supplementation on obesity status and related metabolic profiles in participants who received thylakoid supplements for 12 weeks [16, 18]. All the participants received a calorie-restricted diet designed by a dietitian. For planning the calorie-restricted diet, total energy expenditure was calculated based on resting energy expenditure (REE), which was calculated based on the Mifflin equation [22], physical activity level, and thermic effect of food (10% of total energy expenditure). After calculating the daily required energy for each participant, a calorie deficit of 500 kcal per day was made for each person. Macronutrient distribution was organized as 30, 15, and 55% of energy from fat, protein, and carbohydrates, respectively. This distribution of macronutrients, as moderate and conventional ratios instead of higher or lower ratios (such as low fat or low carbohydrate or high protein), was planned based on existing clinical nutrition guidelines and aimed to increase adherence to the prescribed diet [23]. The participants were asked to pursue healthy eating recommendations, including substituting solid fats and animal fats with non-hydrogenated vegetable oils as well as limiting added sugar, desserts, sugar-sweetened beverages, saturated and trans fats, and fast foods as well as changing cooking methods to healthier ways. The application of food exchange lists was thoroughly explained to the participants, and they were advised of the alternative options with equal calories when a particular item from the corresponding food group is not affordable or available. Adherence to the recommended diet was evaluated using dietary intake records for 3 days (2 weekdays and 1 weekend) at the beginning, middle, and end of the study. Daily intakes of macro- and micro-nutrients were calculated by analyzing food data using nutritionist IV software (First Databank, San Bruno, CA).

### Preparation of spinach thylakoids and placebo

Fresh baby spinach leaves (*Spinacia oleracea*) were used to prepare of thylakoid membranes according to the previously registered protocols [17, 24, 25]. The required spinach was collected from Tabriz, East Azerbaijan Province, Iran in spring, 2018; some plant samples were delivered to the Herbarium Center of the Faculty of Pharmacy, Tabriz University of Medical Sciences. The scientific name of the collected specimen is *Spinacia oleracea* L. belonging to the Oleracea family with the herbarium number TBZ-fph-1898. The thylakoid supplement used in this investigation was prepared based on the method described by Emerk et al. [25], at an experimental scale in the Synthesis Laboratory of Drug Applied Research Center, Tabriz University of Medical Sciences. Fresh spinach leaves after removing the stems and veins, were soaked in cold water and washed. Spinach leaves (1000 g) were homogenized with 1250 ml water in a blender and filtered through four layers of Monodur polyester mesh (20 μm). This obtained filtrate was diluted 10 times with distilled water, and its pH adjusted to 4.7 with Hydrochloric acid (HCl). PH 4.7 is the isoelectric point of the thylakoids, and maximum precipitation occurs at this pH. The thylakoids flocculated, and a green precipitate with a clear, a bit yellowish supernatant was obtained after 4 h standing in the cold (− 4 °C). The supernatant was removed, and the green precipitate was collected from the filtrate thylakoids at pH 4.7 and washed in water by repeated centrifugation; the precipitation was repeated at the same pH. The washed thylakoids were collected, and after adjusting to the desired pH (pH 7.0), the final sediments freeze-dried to obtain a green thylakoid powder. Large scale production, of this freeze-dried thylakoid powder, was conducted by the Iran Darook Pharmaceutical Co., Tehran, Iran. Placebo consisted of corn starch, which was colored in edible green color, and like thylakoid powder, flavored with kiwifruit essence. Corn starch, a white, tasteless, safe, non-toxic, non-irritant, and non-allergenic odorless powder, is the most frequently used substance in the food and pharmaceutical industries, as an inert (inactive) substance without any therapeutic effect. Due to its versatility and flexibility in application, and ease of modification, we modified it to green powder with Kiwi odor, exactly like that extracted thylakoid from spinach. Therefore, green powder of thylakoid or placebo was made, with kiwifruit flavor and identical appearances (shape, size, and color). Next, they were packed in identical sachets with each sachet containing 5 g of thylakoid or 5 g of cornstarch powder. The contents of the sachets were dissolved in a glass of water and consumed 30 min before lunch. Packages were coded and distributed monthly by a third person who was not involved in any other aspects of the study. To remind participants to consume their supplements, a supplement consumption chart was provided. This chart was required to be returned at each visit to ensure compliance. Participants received a daily text message reminder and weekly a phone call reminds them to take the supplement. This helped to minimize study withdrawal and ensure adherence to the study protocol. The participants were asked to return the remaining sachets at each visit; counting these sachets allowed us to evaluate compliance. Consuming ≥80% of the supplements was considered compliant.

### Anthropometric and body composition measurements

The height, weight, waist and hip circumferences, and body composition of the participants were measured at baseline, mid, and end of the study. BMI was calculated as weight (kg) divided by height in meters squared (m^2^). Body composition was assessed using Tanita MC-780 S MA (Amsterdam, the Netherlands). Tanita MC-780 MA is a body composition analyzer based on bioelectrical impedance analysis, which uses BIA technology, as quick, non-invasive, and one of the most thorough and reliable ways, to measure body composition [26].

### Blood sampling and biochemical measurements

At pre- and post-intervention, a 10 ml venous blood sample was drawn from each participant. Blood sampling was done between 8: 00 a.m. and 9:00 a.m. after a 12-h overnight fasting and resting in bed during the early follicular phase (d 2–5) of a spontaneous or P-induced menstrual cycle. Blood samples immediately centrifuged at 3500 rpm for 10 min, and serum samples were separated from whole blood and were frozen immediately at − 80 °C until assay time. Measurements of serum non-esterified fatty acids (NEFA), FBG, and insulin were done on the sampling day. Serum FBG and NEFA were measured through enzymatic methods using the colorimetric technique, by commercial kits (Pars-Azmoon Co., Tehran, Iran; and Biovision Research Product, USA, respectively) by an auto-analyzer (Hitachi-917, Tokyo, Japan). Serum insulin level was measured by chemiluminescence (IMMULITE 2000, SIEMENS); and the homeostatic model of assessment for insulin resistance (HOMA-IR) and β cell function (HOMA-B) and quantitative insulin sensitivity check index (QUICKI) were calculated on the basis of suggested formulas [27]. Hormonal profiles including LH, FSH, dehydroepiandrosterone sulfate (DHEAS), testosterone, and SHBG, were determined using ELISA kits (Bioassay Technology Laboratory, Shanghai Korean Biotech, Shanghai City, China) according to the manufacturer’s instructions with inter- and intra-assay coefficient variances (CVs) lower than 7%. The free androgen index (FAI) was calculated based on suggested formulas [28].

### Statistical analysis

All statistical analyses were performed using SPSS version 23 (SPSS Inc., Chicago, IL, USA). The Kolmogorov-Smirnov test was performed to determine the normality of data distribution. Distribution of data was expressed as mean (SD) for normally distributed quantitative data, and frequency (percent) for qualitative data. To compare the two groups at the baseline, independent sample t-tests, Mann–Whitney, and chi-squared tests were used. Assessments of differences within the group were made by paired-samples t-tests or nonparametric Wilcoxon signed-rank test, and sign test. A comparison of the two groups at the end of the study was completed by the analysis of covariance (ANCOVA) after adjusting for the baseline parameters and covariates (changes in baseline values of evaluated parameters and potential covariates including age, baseline BMI, and the changes in weight, calorie intake, and physical activity level during the study). Post hoc paired comparisons were made by using a Sidak test. Results with *P* values of < 0.05 were considered statistically significant.

## Results

### Baseline characteristics of participants

**Fig. 1:**
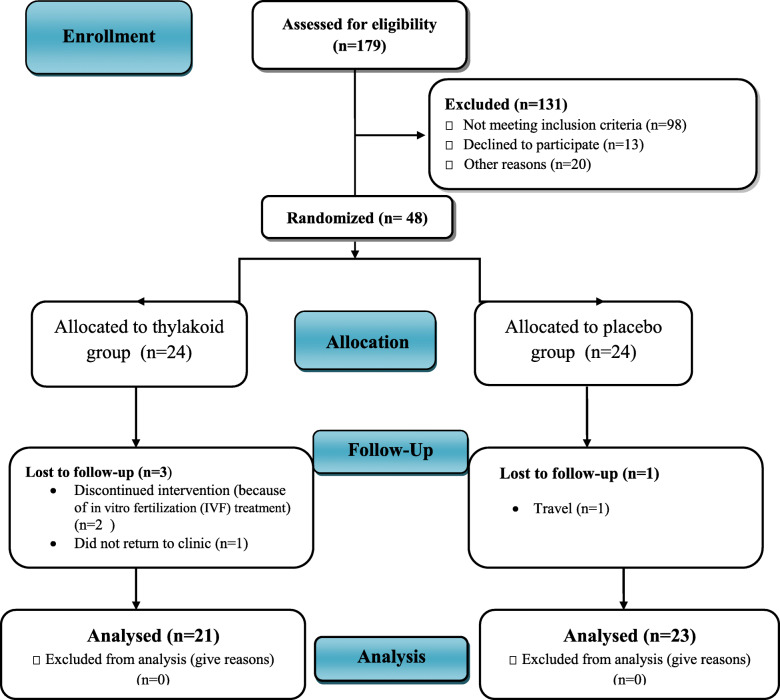
Follow of participants throughout the intervention (Study flow diagram)

Forty-eight obese women with PCOS were recruited in the present clinical trial and randomized to receive either thylakoid or placebo; out of them, forty-four participants [thylakoid (*n* = 21) and placebo (*n* = 23)] completed the trial, while 4 participants discontinued the study for reasons irrelevant to the interventions (Fig. 1). During the trial, no adverse events or symptoms were reported following the intake of thylakoid powder by the participants. Sachet counts showed good compliance, with more than 90% of sachets taken during the course of the trial by both groups. No significant differences were seen in baseline parameters between the two groups (Table 1; *P* > 0.05). None of the participants were smokers. Physical activity levels of the participants did not change significantly throughout the study within or between the two groups (*P* > 0.05).

**Table 1:** Baseline characteristics of the study participants

Variable	Thylakoid group (***n*** = 21)	Placebo group (***n*** = 23)	P
**Age (y)**^**d**^	31.86 (2.35)	32.04 (2.83)	0.81^a^
**Age at menarche (y)** ^**d**^	12.40 (0.46)	12.33 (0.45)	0.59 ^b^
**Height (cm)** ^**d**^	159.33 (3.5)	158.00 (3.4)	0.21^a^
**Physical activity** ^**e**^			1.00 ^c^
***Low***	19 (90.5)	20 (87.0)	
***Moderate***	2 (9.5)	3 (13.0)	
***High***	0 (0.0)	0 (0.0)	

^d^ Values are presented as mean (SD)

^e^ Values are presented as number (%)

^a^ *P* based on Independent-samples t-test

^b^ *P* based on Mann-Whitney U test

^c^ *P* based on chi square test

### Dietary intakes and anthropometric measurements

As shown in Table 2, the baseline calorie and macronutrient intakes were not significantly different in the two groups (*P* > 0.05), except for saturated fatty acids (SFA) and monounsaturated fatty acids (MUFA) intakes, which were higher in the thylakoid group than the placebo group (*P* < 0.05). Both groups received a calorie-restricted diet; therefore, energy intake decreased in the two study groups compared to the baseline. At the end of the study, there were significant decreases in the intake of calories, carbohydrates, total fat, polyunsaturated fatty acids (PUFA), SFA, and MUFA in both thylakoid and placebo groups (*P* < 0.05). Intakes of protein, fiber, and cholesterol did not significantly change in both groups (*P* > 0.05). The between-group analyses adjusted for baseline measures and confounding factors showed no significant differences for these dietary intake parameters (Table 2). Regarding macronutrient distribution (as a percentage of total calorie intake), there were significant decreases in the percentage of calorie from carbohydrates (55% vs. 56.8% in the thylakoid group; 55% vs. 57% in the placebo group; *p* < 0.001 for both groups) and fats (30.2% vs. 32.4% in the thylakoid group; 30.1% vs. 32.2% in the placebo group; *p* < 0.001 for both groups). However, the percentage of total calorie from protein was significantly increased by the end of the study (15% vs. 10.8% in the thylakoid group; 15% vs. 10.7% in the placebo group; p < 0.001 for both groups). There was no significant difference between the two groups in terms of the changes in macronutrients distribution throughout the study.

**Table 2:** Anthropometric measurements and dietary intakes of the study participants at the baseline and after 12 weeks of intervention

		Thylakoid group (*n* = 21)	Placebo group (*n* = 23)	MD (95% CI), ***P***
**Weight (kg)**	Baseline	89.21 (6.50)	88.14 (7.27)	1.06 (− 3.14, 5.27), 0.612**
	End	82.23 (6.16)	84.95 (6.87)	−3.71 (− 4.03, − 3.39), < 0.001***
	MD (95% CI), P*	−6.97 (−7.21, − 6.73), **< 0.001**	− 3.19 (− 3.50, − 2.88), **< 0.001**	
**BMI (kg/m**^**2**^**)**	Baseline	35.13 (2.16)	35.31 (2.77)	− 0.18 (− 1.71, 1.34), 0.808**
	End	32.38 (2.08)	34.03 (2.60)	− 1.47 (− 1.59,- 1.35), < 0.001***
	MD (95% CI), P*	−2.74 (− 2.82, − 2.66), **< 0.001**	−1.28 (− 1.41, − 1.15), **< 0.001**	
**WC (cm)**	Baseline	108.09 (3.89)	108.18 (4.22)	− 0.08 (− 2.56, 2.39), 0.944**
	End	100.31 (4.58)	104.46 (3.75)	−4.00 (− 5.25, − 2.76), < 0.001***
	MD (95% CI), P*	−7.78 (−8.92, − 6.65), **< 0.001**	−3.73 (− 4.33, − 3.12), **< 0.001**	
**WHR**	Baseline	0.92 (0.01)	0.93 (0.01)	−0.01 (− 0.02, − 0.001),0.022**
	End	0.87 (0.03)	0.92 (0.02)	−0.04 (− 0.05,- 0.03), < 0.001***
	MD (95% CI), P*	−0.06 (− 0.06, − 0.04), **< 0.001**	−0.02 (− 0.02, − 0.01), **< 0.001**	
**FM (kg)**	Baseline End MD (95% CI), P*	32.40 (3.46) 27.21 (3.31) −5.19 (− 5.43, −4.94), **< 0.001**	32.17 (3.90) 30.82 (3.90) −1.35 (− 1.52, − 1.18), **< 0.001**	0.22 (− 2.03, 2.47), 0.843** −3.79 (− 4.08, − 3.51), < 0.001***
**FFM (kg)**	Baseline End MD (95% CI), P*	56.79 (3.21) 55.01 (3.09) − 1.78 (− 1.94, − 1.62), **< 0.001**	55.96 (3.53) 54.13 (3.13) − 1.83 (− 2.11, − 1.56), **< 0.001**	0.82 (− 1.23, 2.88), 0.422** 0.07 (− 0.20, 0.34), 0.613***
**Energy (Kcal)**	Baseline	2346.3 (399.96)	2297.8 (446.26)	48.46 (− 210.31, 307.24), 0.707**
	End	1749.37 (117.60)	1711.3 (119.84)	− 0.002 (− 0.004, 0.000), 0.129***
	MD (95% CI), P*	− 596.96 (− 791.85, − 402.08), **< 0.001**	− 586.55 (− 790.50, − 382.60), **< 0.001**	
**Carbohydrates (g)**	Baseline	333.24 (57.00)	326.82 (62.21)	6.41 (−30.00, 42.83), 0.724**
	End	240.54 (16.17)	235.31 (16.48)	2.14 (−2.51, 6.79), 0.357***
	MD (95% CI), P*	−92.70 (− 120.55, − 64.85), **< 0.001**	− 91.52 (− 119.64, − 63.39), **< 0.001**	
**Carbohydrates (%Kcal)**	Baseline End MD (95% CI), P*	56.85 (1.25) 55.01 (0.01) −1.84 (−2.41, − 1.27), **< 0.001**	56.99 (1.62) 55.04 (0.03) − 1.99 (− 2.69, − 1.28), **< 0.001**	− 0.14 (− 1.03, 0.74), 0.75** 0.00 (0.00, 0.00), 1.00***
**Protein (g)**	Baseline	63.72 (13.93)	62.48 (15.56)	1.23 (−7.78, 10.25), 0.784**
	End	65.60 (4.41)	64.17 (4.49)	0.69 (−0.05, 1.44), 0.066***
	MD (95% CI), P*	1.88 (−4.92, 8.69),0.570	1.69 (−5.42, 8.80),0.627	
**Protein (%Kcal)**	Baseline End MD (95% CI), P*	10.78 (0.50) 15.00 (0.00) 4.21 (3.98, 4.44), **< 0.001**	10.77 (0.58) 15.01 (0.00) 4.23 (3.97, 4.48), **< 0.001**	0.012 (−0.32, 0.34), 0.94** 0.00 (0.00, 0.00), 1.00***
**Fat (g)**	Baseline	84.28 (13.73)	82.30 (16.00)	1.98 (−7.12, 11.09), 0.662**
	End	58.31 (3.92)	57.04 (3.99)	0.78 (−0.75, 2.32), 0.308***
	MD (95% CI), P*	−25.97 (−32.56,-19.37), **< 0.001**	− 25.25 (− 32.64, −17.86), **< 0.001**	
**Fat (%Kcal)**	Baseline End MD (95% CI), P*	32.37 (1.30) 30.20 (0.03) − 2.37 (− 2.96,-1.77), **< 0.001**	32.24 (1.58) 30.11 (0.00) − 2.13 (− 2.92,-1.55), **< 0.001**	0.13 (− 0.75, 1.01), 0.76** 0.00 (0.00, 0.00), 1.00***
**Cholesterol (mg)**	Baseline	202.70 (39.18)	198.20 (44.45)	4.49 (− 21.10, 30.10), 0.725**
	End	200.39 (27.74)	190.62 (37.42)	8.49 (−8.34, 25.32), 0.313***
	MD (95% CI), P*	−2.30 (−19.07, 14.46), 0.777	−7.58 (− 20.59, 5.44), 0.240	
**MUFA (g)**	Baseline	20.15 (3.29)	17.20 (3.33)	2.95 (0.93, 4.97), 0.005**
	End	13.93 (0.86)	11.91 (0.78)	−0.26 (− 0.61, 0.09), 0.143***
	MD (95% CI), P*	−6.22 (−7.79, −4.65), **< 0.001**	−5.28 (− 6.83, −3.74), **< 0.001**	
**PUFA (g)**	Baseline	41.85 (6.99)	42.81 (8.42)	− 0.96 (−5.70, 3.77), 0.685**
	End	28.96 (2.15)	29.68 (2.27)	−0.27 (−1.06, 0.52), 0.491***
	MD (95% CI), P*	−12.89 (− 16.19, −9.60), **< 0.001**	− 13.13 (− 16.98, − 9.28), **< 0.001**	
**SFA (g)**	Baseline End MD (95% CI), P*	15.83 (2.69) 11.04 (1.26) −4.78 (−6.00, − 3.57), **< 0.001**	14.02 (2.74) 9.72 (0.72) − 4.30 (− 5.56, − 3.04), **< 0.001**	1.81 (0.15, 3.47), 0.033** 0.04 (− 0.12, 0.20), 0.585***
**Fiber (g)**	Baseline End MD (95% CI), P*	19.16 (5.52) 21.13 (5.38) 1.97 (− 1.23, 5.16), 0.213	20.96 (3.88) 22.59 (4.54) 1.62 (− 0.52, 3.76), 0.130	−1.81 (− 4.69, 1.08), 0.213** − 0.98 (− 4.16, 2.20), 0.535***

*Abbreviation*: *BMI* body mass index, *WC* Waist circumference, *WHR* waist-to-hip ratio, *FM* fat mass, *FFM* fat free mass, *SFA* saturated fatty acid, *MUFA* monounsaturated fatty acid, *PUFA* polyunsaturated fatty acid; % Kcal, percentage of energy. Data are expressed as mean (standard deviation) and mean difference (95% CI). P*: Denotes the significance of within-group changes (Paired samples t- test). P**: P values indicate comparison between groups at baseline (Independent sample t-test for baseline). P***: *P* values indicate comparison between groups based on ANCOVA adjusted for baseline measures and confounding factors (age, baseline BMI, and the changes in weight, calorie intake, and physical activity level during the study)

**Fig. 2:**
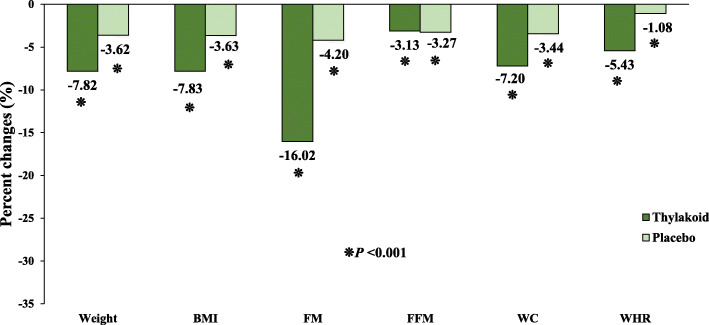
Percent changes in anthropometric measures in thylakoid and placebo groups after 12 weeks’ intervention. *P* values obtained from intra group-paired analyses: the paired t-test. BMI: Body mass index, WC: Waist circumference, WHR: Waist-to-hip ratio, FM: Fat mass, FFM: Fat free mass

As presented in Table 2, at the start of the study, no significant differences were seen in the baseline anthropometric indices including, weight, BMI, waist circumferences (WC), fat mass (FM), and fat-free mass (FFM), except in waist to hip ratio (WHR), between the two groups. After 12 weeks of intervention, changes in weight, BMI, WC, WHR, FM, and FFM were found to be significant and decreased in both study groups (*P* < 0.05). Based on the between-group analysis adjusted for baseline values and changes in dietary intakes and physical activity levels, thylakoid intake combine with a calorie-restricted diet led to significant decreases in weight, BMI, WC, WHR, and FM with no significant changes in FFM, compared to the placebo group (Table 2). Percent changes (PCs) for the anthropometric variables are presented in Fig. 2.

### Glycemic indices and hormonal parameters

Table 3 presents the glycemic indices and hormonal parameters of subjects at baseline and after the intervention. There were no significant differences in levels of FBG, insulin, NEFA, HOMA-IR, HOMA-β, QUICKI, and hormonal parameters between the two groups at baseline (*P* > 0.05). After 12 weeks of intervention, the serum concentrations of FBG and FSH did not change statistically in the two groups compared to baseline values (for both variables *P* > 0.05). In addition, at the end of the study, these parameters were not statistically different between the groups (*P* > 0.05). Changes in the HOMA-IR, HOMA-B, QUICKI, and fasting serum levels of insulin, NEFA, LH, total testosterone, and FAI were significant in both thylakoid and placebo groups compared to baseline values (all *P* < 0.05). At the end of the study, the levels of DHEA-S decreased, and SHBG increased significantly in the thylakoid group compared to baseline values (for both variables *P* < 0.05). As shown in Table 3, results of the analysis of covariance after adjusting for the baseline values and potential confounding factors (changes in weight, physical activity, and calorie intake) revealed no significant between-group differences by the end of the study for these parameters, except for levels of insulin, total testosterone, HOMA-IR, HOMA-B, and QUICKI. Comparison between two groups indicated that the QUICKI levels increased, and levels of insulin, total testosterone, HOMA-IR, and HOMA-B decreased significantly in the thylakoid group compared to the placebo group (all *P* < 0.05). Accordingly, the between-group analysis showed that these parameter levels were improved significantly in the thylakoid treated group (*P* < 0.05).

**Table 3:** Comparison of metabolic and hormonal parameters between the thylakoid group and the placebo group at baseline and after the intervention

		Thylakoid group (n = 21)	Placebo group (n = 23)	MD (95% CI), ***P***
**FBG (mg/dl)**	Baseline	93.09 (5.39)	96.56 (7.44)	− 3.47 (− 7.46, 0.52), 0.086**
	End	92.66 (4.70)	96.09 (6.10)	− 0.28 (− 3.35, 2.79), 0.854***
	MD (95% CI), P*	−0.43 (−1.05, 0.19), 0.165	−0.48 (−1.36, 0.40), 0.273	
**Insulin (**μU**/mL)**	Baseline	17.97 (2.45)	18.63 (2.33)	− 0.65 (− 2.11, 0.80), 0.370**
	End	12.57 (2.11)	17.43 (2.25)	−3.73 (− 7.06,- 0.40), 0.029***
	MD (95% CI), P*	− 5.40 (− 6.25, − 4.55), **< 0.001**	−1.19 (− 1.56, − 0.83),**< 0.001**	
**HOMA-IR**	Baseline	4.14 (0.69)	4.43 (0.60)	−0.29 (− 0.68, 0.10), 0.143**
	End	2.87 (0.49)	4.13 (0.53)	−1.06 (− 1.88,-0.25), 0.011***
	MD (95% CI), P*	−1.27 (− 1.48, − 1.05), **< 0.001**	−0.31 (− 0.42, − 0.19),**< 0.001**	
**HOMA-B**	Baseline	219.68 (40.36)	210.36 (55.60)	9.32 (−20.48, 39.13),0.531**
	End	156.06 (37.50)	197.36 (49.16)	− 40.82 (− 78.48, −3.16), 0.034***
	MD (95% CI), P*	−63.62 (− 73.88, − 53.37), **< 0.001**	−13.00 (− 17.53, − 8.46), **< 0.001**	
**QUICKI**	Baseline End MD (95% CI), P*	0.31 (0.007) 0.33 (0.008) 0.02 (0.014, 0.02), **< 0.001**	0.31 (0.006) 0.31 (0.005) 0.003(0.002, 0.004), **< 0.001**	0.003 (−0.002, 0.007), 0.121** 0.012 (0.002, 0.022), 0.022***
**NEFA (mmol/L)**	Baseline End MD (95% CI), P*	0.89 (0.06) 0.86 (0.06) −0.03 (− 0.03, − 0.02), **< 0.001**	0.93 (0.07) 0.90 (0.06) − 0.02 (− 0.03, − 0.01), **< 0.001**	−0.03 (− 0.07, 0.005), 0.084** 0.008 (− 0.033, 0.048), 0.707***
**LH (mIu/ml)**	Baseline	7.99 (0.42)	7.78(0.38)	0.21 (−0.03, 0.45), 0.089**
	End	7.95 (0.42)	7.74 (0.36)	−0.13 (− 0.315, 0.059), 0.173***
	MD (95% CI), P*	−0.04 (− 0.07, − 0.006),**0.022**	−0.04 (− 0.08, − 0.006), **0.025**	
**FSH (mIu/ml)**	Baseline	4.49 (0.05)	4.53 (0.48)	−0.05 (− 0.35, 0.25), 0.751**
	End	4.47 (0.05)	4.52 (0.47)	0.024 (−0.08, 0.13), 0.652***
	MD (95% CI), P*	−0.01 (− 0.03, 0.004), 0.124	− 0.01 (− 0.03, 0.005), 0.146	
**DHEA-S (μg/dl)**	Baseline	246.09 (9.58)	242.56 (7.42)	3.53 (−1.66, 8.72), 0.177**
	End	241.66 (5.65)	241.91 (5.88)	2.87(−10.72, 16.47), 0.671***
	MD (95% CI), P*	−4.43(−8.64, −0.22),**0.040**	−0.65(−4.52, 3.21),0.730	
**Total testosterone (ng/ml)**	Baseline	0.65 (0.06)	0.69 (0.08)	−0.03 (− 0.08, 0.01), 0.121**
	End	0.59 (0.03)	0.66 (0.07)	−0.06 (− 0.12,- 0.004), 0.036***
	MD (95% CI), P*	−0.06 (− 0.08,-0.04), **< 0.001**	−0.03 (− 0.03, − 0.02), **< 0.001**	
**SHBG (nmol/L)**	Baseline	25.83 (1.12)	25.76 (1.02)	0.72 (−0.58, 0.72), 0.824**
	End	26.28 (1.13)	25.97 (0.79)	−0.52 (−2.02, 0.97), 0.483***
	MD (95% CI), P*	0.45 (0.06, 0.84), **0.026**	0.22 (−0.01, 0.44), 0.057	
**FAI**	Baseline	8.82 (0.98)	9.28 (1.07)	−.46 (−1.09, 0.16), 0.143**
	End	7.87 (0.48)	8.83 (0.94)	−0.67 (−1.5, 0.19), 0.121***
	MD (95% CI), P*	−0.95 (−1.25, − 0.66), **< 0.001**	−0.45 (− 0.58, − 0.32), **< 0.001**	

*DHEAS* Dehydroepiandrosterone sulfate, *FAI* free androgen index, *FBG* fasting blood glucose, *FSH* Follicle stimulating hormone, *HOMA-IR* homeostatic model assessment for insulin resistance, *LH* Luteinizing hormone, *NEFA* non-esterified fatty acids (or free fatty acids), *QUICKI* quantitative insulin sensitivity check Index, *SHBG* sex hormone-binding globulin. Mean (SD) and mean difference (95% CI) are presented for data.* P based on Paired samples t-test.** P based on Independent samples t-test. *** P based on ANCOVA adjusted for baseline values and confounding factors (age, baseline BMI, and the changes in weight, calorie intake, and physical activity level during the study)

## Discussion

The findings of previous animal studies and clinical trials support the appetite-suppressing and weight-reducing properties of a thylakoid-rich spinach extract. These studies reported that thylakoid-rich spinach extract might help in the management of obesity and its related metabolic issues such as glycemia and insulin imbalances [17, 19, 29]. Due to the lack of similar studies on obese women with PCOS, the present double-blind placebo-controlled RCT was designed to assess the effects of caloric restriction in combination with thylakoid-rich spinach extract supplementation on the anthropometric measures, metabolic, and hormonal status in obese women suffering from PCOS. The current study, as the first clinical trial in this regard, showed that administration of 5 g/day of thylakoid-rich spinach extract in combination with calorie-restricted diet for 12 weeks in obese women with PCOS resulted in significant reductions in weight, BMI, WC, WHR, and FM compared to placebo. Meanwhile, these anthropometric indices significantly decreased at the end of the study in both thylakoid and placebo groups, which could be due to receiving the calorie-restricted diet; however, between-group differences were significant at the end of the study. These findings are in line with earlier studies [17, 30, 31]. In a 12-week single-blinded RCT, thylakoids were shown to promote body weight loss significantly when compared to controls among overweight or obese women [18]. In this study, consumption of 5 g of thylakoid/day resulted in a significant reduction (6.3%) in body weight compared to the placebo group. However, there were no significant differences in WC, FFM, and body fat percentage between the two groups. Our findings showed that coupled with weight loss, a reduction in body fat mass was the most significant change induced by thylakoids compared to controls. Further, changes in FFM were not significant between the two groups. The findings of a study on an animal model of obesity (Female Sprague-Dawley rats fed a high-fat diet) indicated that oral administration of thylakoids resulted in significantly less food intake over the 13-day study and significantly lower body weights(− 18%) compared to the controls [17]. A longer (100 days) trial confirmed the anti-obesity effect of thylakoid supplementation, which lowered food consumption, body fat (− 33%), and weight (− 27%) in female apo E-deficient mice [32]. Several explanations have been suggested for the positive effects of spinach-derived thylakoids on obesity management. For example, it was demonstrated that thylakoid membranes isolated from spinach inhibited the activity of pancreatic lipase/co-lipase up to 80% in vitro [17]. Thus, thylakoids slow down the digestion and absorption of dietary fat; hence, post-prandial high-fat content in the small intestinal lumen curbs hunger, stimulates satiety signals, and promotes satiety and suppresses appetite [33]. Elevated gut-derived satiety hormone cholecystokinin (CCK) from duodenal enteroendocrine cells following consumption of a thylakoid containing meal, has been reported by Köhnke et al. in healthy humans [30]. In this regard, numerous human trials have been conducted with favorable results on the satiety inducing effects of spinach thylakoids [16, 18, 31, 34]. Overall, significantly elevated levels of the enteroendocrine-derived satiety hormone glucagon-like peptide-1(GLP-1) (+ 44%) and the adipose-derived satiety hormone leptin were demonstrated. Further, a significant reduction was reported in the serum levels of ghrelin, a stomach-derived hunger hormone, 2-h after consuming a thylakoid-enriched meal [18]. The promotion of satiety as well as suppression of hunger and appetite by thylakoid has been highlighted by previous studies regarding weight loss and anthropometrics improvement effects of spinach-derived thylakoids [35]. Nevertheless, our findings showed that thylakoid supplementation did not alter the energy and dietary nutrient intake in obese women with PCOS. Also, there were no significant differences in energy and dietary intakes between the two groups. Our findings were consistent with the outcomes provided by previous studies [18, 31], which indicated no significant changes in total calorie and nutrient intakes of thylakoid consumers in trials; and the food intake at the next meal following thylakoid intake was not different either between placebo and thylakoid groups. Thus, the exact mechanism of thylakoid affecting anthropometric indices is still unknown. Based on the results of the present clinical trial, 5 g/day of spinach-derived thylakoid supplementation for 12 weeks among obese women with PCOS resulted in significant reductions in insulin levels, HOMA-IR, and HOMA-B. It also significantly elevated QUICKI when compared to the placebo. QUICKI has been suggested as the most extensively validated and accurate surrogate index of insulin sensitivity, so the elevation of this index is indicative of an increase in insulin sensitivity [36]. However, the changes observed in serum levels of FBG and NEFA did not differ significantly between the two groups. Our findings concurred with the findings of Kohnke et al. [30]. They reported that insulin levels were reduced by about 37% in the thylakoid group compared to the control, but blood glucose level changes were not different between the two groups. Significant reductions in insulin levels in thylakoid consumers compared to controls, without considerable differences in glucose level variations between the two groups, suggested increased insulin sensitivity due to thylakoid supplementation. Given the body weight and fat mass reducing effects of thylakoid supplementation in our study, it is expected that thylakoid intake could improve insulin sensitivity and alleviate insulin resistance, as has been demonstrated in other studies [18, 31]. Furthermore, a number of animal studies reported decreased blood glucose levels and improved glucose homeostasis during treatment with thylakoid [24, 32, 37, 38]. These positive effects of spinach-derived thylakoids on glucose homeostasis parameters may be explained by various mechanisms, such as delaying gastric emptying, increasing CCK and GLP-1 secretions, and reducing intestinal uptake of glucose through localization of thylakoids as large complex structures onto the mucosa as well as by binding to the starch and/or amylase, which was observed in rat intestine [24, 37]. Further, the loss of both body weight and fat mass due to thylakoid supplementation could be involved in these beneficial metabolic effects. A loss of body weight is often associated with an increase in the levels of free fatty acids (FFAs). FFAs reduce insulin sensitivity in muscles by inhibiting insulin-mediated glucose uptake. An overflow of circulating FFAs, released from an expanded adipose tissue mass, is the main contributor to the progression of insulin resistance [39]. Several studies have shown a reduction in serum FFAs levels following spinach thylakoid supplementation [30, 32]. Our results show that despite the significant weight loss, FFAs levels did not increase in either groups, and at the end of the study, the differences in FFAs level changes were not significant between the groups. Similar results were observed in some human studies, reporting no effect of thylakoid supplementation on serum FFAs levels [18, 31]. It seems that a greater improvement in insulin resistance and thus improvement of fatty acid and glucose metabolism might have masked the potentially altered levels of FFAs due to loss of weight and fat mass in our participants. Further, differences in the dosage used or duration of supplementation might have played a role in the studies inconsistent outcomes. We found that 5 g/day of thylakoid-rich spinach extract administration in combination with calorie-restricted diet for 12 weeks among obese women with PCOS led to insignificant changes in the hormonal parameters except for total testosterone level which decreased significantly in the thylakoid group, based on between-group analysis adjusted for baseline values and confounders. To the best of our knowledge, no reports are available indicating the effects of spinach thylakoid supplementation on hormonal status in women with PCOS. Regarding the use of oral contraceptives by all participants to help regulate their irregular menstrual cycle, it is expected that the hormonal profile of these subjects would not change significantly due to thylakoid supplementation. The main outcome of the current study is that spinach thylakoid combined with the calorie-restrictive diet was more effective in reducing testosterone levels than did a calorie-restrictive diet alone. The possible explanations for this effect can be found in the greater reductions of weight and fat mass, as well as greater improvement of insulin resistance in thylakoid consumers on the calorie-restrictive diet, compared to the placebo group. Previous studies have established that calorie restriction and weight loss can reduce insulin levels and improve insulin sensitivity through enhancing adenosine monophosphate-activated protein kinase (AMPK) signaling and affecting the release of inflammatory mediators from visceral adipose tissue [40, 41]. In our study, all participants were obese and insulin-resistant, and both groups received a calorie-restrictive diet. Nevertheless, we found that the reductions in weight, fat mass, and WC in the thylakoid receiving group were significantly lower compared to the placebo receiving group. Thus, because of the interrelated interactions between IR, hyperandrogenism, and obesity, a possible reason for beneficial changes in insulin resistance and serum levels of insulin and testosterone might in part be attributed to the improvement of anthropometric parameters, following spinach thylakoid supplementation in our patients.

The major strength of our trial, as the first clinical trial, was monitoring the patients’ diets by designing calorie-restricted dietary plans with a focus on the amount and type of the macronutrients, and individual preferences, which enhanced compliance. Further, physical activities and dietary intakes were checked before, during, and after the intervention. Furthermore, frequent visits and phone calls increased the participants’ motivation and cooperation. The design of our study, double-blind and the adjustment of measured biochemical parameters for the known confounding factors were the principal strengths of this study. However, the present trial had some limitations including small sample size, the absence of a follow-up evaluation of ovarian masses via sonographic assessment, and not testing for a dose-response relationship between thylakoid intake and appeared changes in the measured parameters. The use of the bioelectrical impedance analysis to assess body composition was the other limitation of this trial. Impedance instruments measure the body’s electrical impedance, and the measured impedance is then used to predict body composition. Thus, body composition is indirectly predicted from impedance due to the involvement of assumptions and estimations in this process. Further, BIA measures are dependent on several factors, such as age, gender, ethnicity, the presence of medical conditions, and hydration status. Hydration or dehydration, exercise, menstruation, consuming alcohol or caffeine, and having a full bladder can all affect the precision of BIA measurements [42].

## Conclusions

The present study, for the first time, reported that supplementation with 5 g/day spinach-derived thylakoid in combination with a calorie-restrictive diet for 12 weeks may improve anthropometric indices, insulin sensitivity, and testosterone levels in obese women with PCOS. These findings highlighted the fundamental importance of weight loss and lowering BMI in the management of metabolic disturbance in obese women with PCOS. Collectively, these data suggest that spinach-derived thylakoid along with calorie-restrictive diet may be a promising adjunct therapy without any side effects for the management of obesity and insulin sensitivity, in OCP treated obese women with PCOS. Yet, further studies are required to identify the exact mechanisms of these beneficial effects and to confirm the positive effects of spinach-derived thylakoids in obese women with PCOS.

## Data Availability

Data that support the findings of this study are available from the corresponding author upon reasonable request.

## Abbreviations

BMI: Body mass index
BIA: Bioelectrical impedance analysis
CCK: Cholecystokinin
CI: Confidence interval
CVs: Coefficient of variances
DHEAS: Dehydroepiandrosterone sulfate
FAI: Free androgen index
FBG: Fasting blood glucose
FFM: Fat free mass
FM: Fat mass
FBS: Fasting blood glucose
FSH: Follicle-stimulating hormone
GLP-1: glucagon-like peptide-1
HOMA-IR: Homeostasis model assessment of insulin resistance
IR: Insulin resistance
LDL-C: Low-density lipoprotein cholesterol
LH: Luteinizing hormone
MUFA: Monounsaturated fatty acid
NEFA: Non-esterified fatty acids
OCP: Oral contraceptive pill
PCOS: Polycystic ovary syndrome
PUFA: Polyunsaturated fatty acid
QUICKI: Quantitative insulin sensitivity check index
RCT: Randomized clinical trial
REE: Resting energy expenditure
SD: Standard deviation
SFA: Saturated fatty acid
SHBG: Sex hormone-binding globulin
WC: Waist circumference
WHR: Waist-to-hip ratio
